# Therapeutic Benefit of the Association of Lodenafil with Mesenchymal Stem Cells on Hypoxia-induced Pulmonary Hypertension in Rats

**DOI:** 10.3390/cells9092120

**Published:** 2020-09-18

**Authors:** Marina de Moraes Carvalho da Silva, Allan Kardec Nogueira de Alencar, Jaqueline Soares da Silva, Tadeu Lima Montagnoli, Grazielle Fernandes da Silva, Bruna de Souza Rocha, Guilherme Carneiro Montes, Rosália Mendez-Otero, Pedro Moreno Pimentel-Coelho, Juliana F. Vasques, Margarete Manhães Trahez, Roberto Takashi Sudo, Gisele Zapata-Sudo

**Affiliations:** 1 Programa de Pesquisa em Desenvolvimento de Fármacos, Instituto de Ciências Biomédicas, Universidade Federal do Rio de Janeiro, Rio de Janeiro, RJ 21941-902, Brazil; marina-mcs@hotmail.com (M.d.M.C.d.S.); allankdc@gmail.com (A.K.N.d.A.); ssjck@hotmail.com (J.S.d.S.); tmontagnoli@gmail.com (T.L.M.); graziellemed@gmail.com (G.F.d.S.); brunadesouzarocha.98@gmail.com (B.d.S.R.); montes.guilherme@gmail.com (G.C.M.); mmtrachez@gmail.com (M.M.T.); rtakashisudo@gmail.com (R.T.S.); 2 Programa de Pós-Graduação em Farmacologia e Química Medicinal, Instituto de Ciências Biomédicas, Universidade Federal do Rio de Janeiro, Rio de Janeiro, RJ 21941-902, Brazil; 3 Programa de Pós-Graduação em Cardiologia, Instituto do Coração Edson Saad, Universidade Federal do Rio de Janeiro, Rio de Janeiro, RJ 21941-913, Brazil; 4 Instituto de Biofísica Carlos Chagas Filho, Universidade Federal do Rio de Janeiro, Rio de Janeiro, RJ 21941-902, Brazil; rmotero@biof.ufrj.br (R.M.-O.); pedrompc@biof.ufrj.br (P.M.P.-C.); julianavasques@biof.ufrj.br (J.F.V.)

**Keywords:** pulmonary arterial hypertension, PDE5 inhibitor, human mesenchymal stem cells, association therapy

## Abstract

Pulmonary arterial hypertension (PAH) is characterized by the remodeling of pulmonary arteries, with an increased pulmonary arterial pressure and right ventricle (RV) overload. This work investigated the benefit of the association of human umbilical cord mesenchymal stem cells (hMSCs) with lodenafil, a phosphodiesterase-5 inhibitor, in an animal model of PAH. Male Wistar rats were exposed to hypoxia (10% O_2_) for three weeks plus a weekly i.p. injection of a vascular endothelial growth factor receptor inhibitor (SU5416, 20 mg/kg, SuHx). After confirmation of PAH, animals received intravenous injection of 5.10^5^ hMSCs or vehicle, followed by oral treatment with lodenafil carbonate (10 mg/kg/day) for 14 days. The ratio between pulmonary artery acceleration time and RV ejection time reduced from 0.42 ± 0.01 (control) to 0.24 ± 0.01 in the SuHx group, which was not altered by lodenafil alone but was recovered to 0.31 ± 0.01 when administered in association with hMSCs. RV afterload was confirmed in the SuHx group with an increased RV systolic pressure (mmHg) of 52.1 ± 8.8 normalized to 29.6 ± 2.2 after treatment with the association. Treatment with hMSCs + lodenafil reversed RV hypertrophy, fibrosis and interstitial cell infiltration in the SuHx group. Combined therapy of lodenafil and hMSCs may be a strategy for PAH treatment.

## 1. Introduction

Pulmonary arterial hypertension (PAH) is a disease characterized by pulmonary artery (PA) wall remodeling and hypertrophy [1], while promoting inflammation [2] and vascular tonus dysfunction [3], which result in an increase in pulmonary arterial pressure [4]. Vascular remodeling leads to overload of the right ventricle (RV), which at first responds with concentric hypertrophy, followed by dysfunction and failure, the major factor for mortality [5].

Drug classes currently approved for PAH treatment focus on promoting pulmonary artery relaxation [6], thus reducing RV overload and improving quality of life [7]. Phosphodiesterase 5 (PDE5) inhibitors approved to treat PAH are sildenafil, tadalafil and vardenafil [7], whose mechanism involves increasing the bioavailability of cyclic guanosine monophosphate (cGMP) by inhibiting its degradation [8]. Therefore, a PDE5 inhibitor developed in Brazil, lodenafil [9], has shown promising results regarding PAH treatment [10] because of its comparable efficacy to sildenafil in preclinical studies on reverting PAH [11]. Mortality consequent to PAH is high [12] because current therapy fails to reverse the structural and signaling changes in pulmonary arteries (deregulated angiogenesis, high production and release of growth factors, strong resistance to apoptosis, abnormal formation of an inflammatory environment within and surrounding vessel walls) [13] and the RV (ischemia, metabolic dysfunction, fibrosis) [5]. Thus, PAH treatment should ideally consider the prevention or reversion of these alterations [14].

One promising alternative in this regard is the use of mesenchymal stem cells (MSCs), which could interfere with the production and release of paracrine factors with regenerative and immunomodulatory properties [15]. In PAH preclinical models, MSCs or MSC-derived vesicles have demonstrated anti-proliferative and anti-inflammatory effects on pulmonary artery and cardioprotective effects on the RV [14,16,17,18]. Human mesenchymal stem cells (hMSCs) derived from umbilical cords have an advantage compared to bone marrow- and adipose tissue-derived MSCs due to their greater longevity and proliferation in culture [19]. Previous preclinical studies using a monocrotaline-induced PAH model have shown benefit of combined therapy with sildenafil and endothelial progenitor cells [20,21]. However, there are unanswered issues regarding the use of MSCs for hypoxia-induced PAH.

The present work investigated the profile of the association of a PDE5 inhibitor, a drug class already in clinical use for PAH treatment, with hMSCs to identify a better and effective treatment than their use in monotherapy. Thus, the potential beneficial effect of the association lodenafil + hMSCs was evaluated in a hypoxia-induced PAH model in rats.

## 2. Materials and Methods

### 2.1. Drugs and Reagents

Lodenafil carbonate was kindly donated by Cristália Produtos Químicos e Farmacêuticos Ltd.a. (Itapira, SP, Brazil) and was dissolved in dimethylsulfoxide (DMSO) at a concentration of 10 mg/mL. SU5416 was synthetized at Laboratório de Avaliação e Síntese de Substâncias Bioativas (LASSBio^®^, Universidade Federal do Rio de Janeiro). The antibodies used were: 1. alpha smooth muscle actin (α-SMA; c. A2547) from Sigma-Aldrich (St. Louis, MO, USA); 2. tumor necrosis factor alpha (TNF-α; c. ab1793), c-fos (c. ab7963), p38 mitogen-activated protein kinase (p38 MAPK; c. ab7952) and inducible nitric oxide synthase (iNOS; ab15323) from Abcam (Cambridge, MA, USA); and 3. extracellular signal-regulated kinase 1/2 (ERK1/2; c. 9102) and phosphorylated extracellular signal-regulated kinase 1/2 (p-ERK1/2; c. 9101) from Cell Signaling Technology (Danvers, MA, USA). Secondary antibodies goat anti-rabbit IgG (1706515) and goat anti-mouse IgG (1706516) horseradish peroxidase (HRP) conjugated secondary antibodies were obtained from Bio-Rad (Hercules, CA, USA) and goat antimouse/anti-rabbit IgG F(ab’)2-HRP polymer conjugate (414191F) was purchased from Nichirei (Tokyo, Japan).

### 2.2. Isolation and Immunophenotyping of Umbilical Cord hSMCs (hMSCs)

Umbilical cord hMSCs were isolated and immunophenotyped as described by Alencar et al. [14]. At the day of intravenous injection, hMSCs were defrosted and washed in a DNase I solution (0.6 U/mL, Ambion, Austin, TX, USA) and then 5.10^5^ cells were administered through the caudal vein. The non-treated group received the same volume (300 μL) of the DNase I solution.

### 2.3. Experimental Design

**Figure 1:**
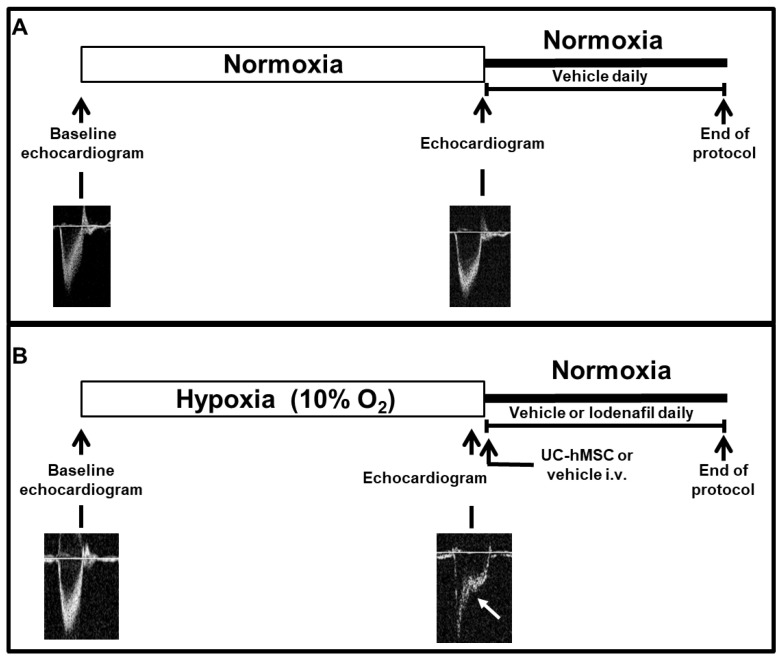
Scheme of the experimental protocol. (**A**) normoxia and (**B**) SuHx-PAH model in rats. PAH, pulmonary arterial hypertension; SuHx, SU5416/hypoxia; UC-hMSCs, umbilical cord human mesenchymal stem cells.

All experiments were approved by the Ethics and Animal Care and Use Committee at the Federal University of Rio de Janeiro (license number 039-19). To induce PAH, Wistar rats (180–250 g) were exposed to normobaric hypoxia (10% O_2_) in a ventilated acrylic chamber controlled by an oxycycler (BioSpherix, Lacona, NY, USA) for 21 days. Together, animals received weekly i.p. administration of SU5416 (20 mg/kg), a vascular endothelial growth factor (VEGF) receptor inhibitor (Figure 1). Control rats were exposed to normoxia and received a weekly i.p. injection of 1 mL/kg of CMC (sodium carboxymethylcelulose 0.5% [*m*/*v*], sodium chloride 0.9% [*m*/*v*], polysorbate 80 0.4% [*v*/*v*], benzilic alcohol in deionized water 0.9% [*v*/*v*]). SU5416/hypoxia-induced PAH (SuHx) provides characteristics similar to the human disease [22]. Doppler pulmonary outflow was recorded to measure the pulmonary artery outflow waveform and confirm PAH establishment when the ratio between pulmonary artery acceleration time (PAAT) and RV ejection time (RVET) was <0.35 [23]. After PAH confirmation, SuHx rats were randomly divided into four groups:

1. SuHx + vehicle (DMSO p.o.);
2. SuHx + lodenafil (10 mg/kg/day p.o.);
3. SuHx + hMSCs (5.10^5^ cells i.v.);
4. SuHx + lodenafil (10 mg/kg/day p.o.) + hMSCs (5.10^5^ cells i.v.).

hMSCs or the vehicle (DNase I in PBS 0.6 U/mL) were injected in the caudal vein of rats on a single dose after the period of 21 days in a hypoxia environment. Oral administration (gavage) of lodenafil carbonate or DMSO was once a day for 14 days. Echocardiography was repeated 12 h after the end of oral treatment followed by RV catheterization.

### 2.4. Echocardiography

Echocardiography evaluation was done by a unique operator, who was blinded to the experimental groups. Echocardiography was performed in animals under anesthesia by isoflurane (Cristália Produtos Químicos e Farmacêuticos Inc., Itapira, SP, Brazil) (3% inhaled) using a system equipped with a 25 MHz probe and a 15 mm focal length (Vevo 770, Visualsonics, Toronto, ON, Canada). Besides acquisition of Doppler pulmonary outflow, echocardiography was used to obtain RV and left ventricle (LV) parameters such as internal area, and wall thickness used B-mode and M-mode, respectively. Data were collected and analyzed by a blinded experimenter.

### 2.5. RV Catheterization

Under anesthesia by ketamin (80 mg/kg, i.p.) and xilazin (15 mg/kg, i.p.), a pressure–volume catheter (SPR-838, Millar Instruments, Houston, TX, USA) connected to a pressure transducer (MLT884, ADInstruments, Inc., Colorado Springs, CO, USA) was introduced through the jugular vein in the rats. The RV systolic pressure (RVSP) was recorded on a computer for analysis using Lab Chart software (Version 7.0, ADInstruments, Inc.). At the end of the experiment, animals were euthanized, and heart and lungs were removed.

### 2.6. Tissue Harvesting

After removal, heart and lung fractions were either stored at −80 °C or fixed through immersion in formaldehyde 10% solution and embedded in paraffin. RV hypertrophy was evaluated through the determination of the ratio between RV weight and LV plus septum (S) weight (RV/VLV + S), Fulton’s index [14].

### 2.7. Vascular Reactivity of Pulmonary Artery

Isometric tension recording of PA reactivity was used to evaluate the endothelial function integrity [24]. Briefly, pulmonary arteries were placed in chambers filled with a physiological solution composed of (in mM): NaCl 123, KCl 4.7, MgCl_2_ 1.2, KH_2_PO_4_ 1.2, glucose 11.5, NaHCO_3_ 15.5, CaCl_2_ 1.2, bubbled with 95% O_2_/5% CO_2_ and maintained at 37 °C. After a 2 h equilibrium period of 1.5 g resting tension, preparations were exposed to increasing concentrations of phenylephrine (1 nM–10 μM). After the plateau of maximal contraction, the arteries were exposure to increasing concentrations of acetylcholine (ACh; 1 nM–10 μM) to determine the vasodilatory activity and endothelial function. ACh-induced response curves were obtained using Lab Chart software (Version 7.0, ADInstruments, Inc., Sydney, Australia).

### 2.8. Membrane Preparation and Western Blot Analysis

Protein expression was measured as published elsewhere [25]. Briefly, tissues stored at −80 °C were homogenized and prepared for electrophoresis in an acrylamide gel and transferred to nitrocellulose membranes. The membranes were incubated with primary antibody solution overnight, then incubated with secondary antibody solution and had protein bands revealed by chemoluminescence using Image Quant LAS4000 (Cytiva, Marlborough, MA, USA).

### 2.9. Histological and Immunohistochemistry Analysis of Pulmonary Arterioles and RV

Pulmonary arteriole muscularization was evaluated using lung slices (5 μm) rehydrated and exposed for 60 min to bovine serum albumin 5% in phosphate-buffered saline (PBS-BSA) solution. Then, tissues were incubated with primary antibody (1:500 anti-α-SMA in PBS-BSA 1%) for 2 h followed by exposure to the secondary antibody (2:3 Histofine Rat PO (Multi) in PBS). After washout with PBS, slices were stained and exposed to 3,3-diaminobenzidine for 5–10 min, counterstained with hematoxylin. Within each lung, 10–20 pulmonary arterioles with external diameter <50 μm were photographed with a digital camera (Canon A620, USA) coupled to an Axiostar optical microscope (Zeiss, Germany) at 1000× magnification. Vessel medial wall area was expressed as the percentage of the wall portion positively stained with α-SMA relative to the total transversal area.

Tissue fibrosis was evaluated through picrosirius red (PSR) staining. In pulmonary arterioles, 10–20 arterioles (external diameter <50 μm) per lung slice were photographed, under a 1000× magnification and perivascular collagen content was measured as the collagen area surrounding an arteriole normalized by its own transverse area. In the RV, 5–10 fields per section were photographed under 400× magnification and interstitial fibrosis was measured by obtaining the total collagen area per tissue area.

In lungs stained with hematoxylin and eosin (HE), 10–20 pulmonary arterioles (diameter <50 μm) per lung slice were photographed under 1000× magnification and divided by the arteriole area for the perivascular cell count, an indicator of inflammatory infiltrate. The same technique was used in RV sections under 400× magnification.

c-fos content in RV and lungs sections was evaluated using immunohistochemistry after incubation in peroxidase-conjugated secondary antibody for 2 h, followed by DAB staining, and was expressed by the percentage of cardiomyocytes with stained nuclei in fields under 1000× magnification. p38 MAPK, TNF-α and iNOS content evaluation in RV sections was also performed and protein content was expressed by percentage stained area of the total field area under 400× magnification.

### 2.10. Statistical Analysis

For statistical analysis, the one-way ANOVA test was used followed by Tukey’s post hoc test using GraphPad Prism, version 6 (GraphPad, San Diego, CA, USA). Differences were considered statistically significant if *p* < 0.05.

## 3. Results

### 3.1. Lodenafil + hMSCs Reduces Vascular Dysfunction on SuHx-Induced PAH

**Figure 2:**
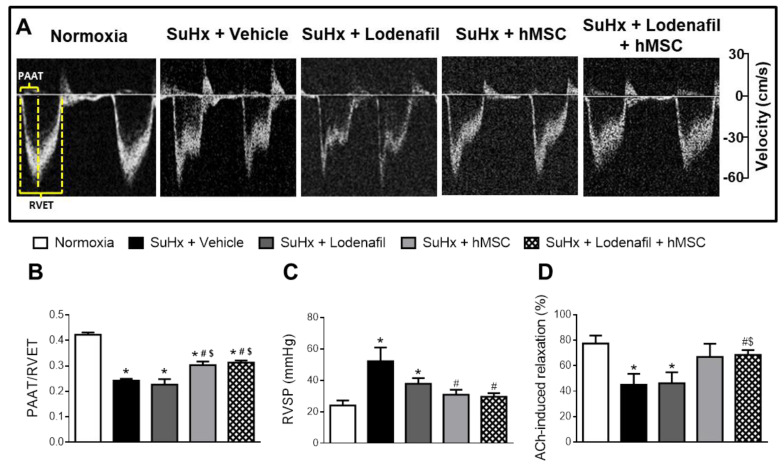
Effects of lodenafil + hMSCs therapy on functional parameters of the cardiovascular system in SuHx-PAH rats. (**A**) Representative images of pulmonary artery ejection flux for each experimental group, obtained using Doppler echocardiography. (**B**) Pulmonary artery acceleration time (PAAT)/right ventricular ejection time (RVET) ratio. (**C**) RV systolic pressure (SP). (**D**) Acetylcholine (Ach)-induced relaxation of pulmonary arteries pre-contracted with phenylephrine (Phe). Data are expressed as mean ± SEM (*n* = 6). * *p* < 0.05 compared to normoxia group; # *p* < 0.05 compared to SuHx group treated with vehicle; $ *p* < 0.05 compared to SuHx group treated with lodenafil. One-way ANOVA with multiple comparisons. ACh, acetylcholine; hMSCs, human mesenchymal stem cells; PAAT, pulmonary artery acceleration time; PAH, pulmonary arterial hypertension; RVET, right ventricle ejection time; RVSP, right ventricle systolic pressure; SuHx, SU5416/hypoxia.

Figure 2A shows the representative pulmonary artery outflow waveform of the different experimental groups obtained by Doppler echocardiography at the end of the protocol. The PAAT/RVET ratio decreased from 0.42 ± 0.01 to 0.24 ± 0.01 (Figure 2B) and monotherapy of lodenafil did not alter this parameter. However, the association of lodenafil + hMSCs partially recovered to 0.31 ± 0.01 (*p* < 0.01), indicating a reduction in the increased pulmonary pressure. RVSP of control rats was of 24.0 ± 3.1 mmHg (Figure 2B), while PAH induced its increase to 52.1 ± 8.8 (SuHx + vehicle) mm Hg. Increased RVSP was reduced by lodenafil and hMSCs to 37.8 ± 3.5 and 30.8 ± 3.2 mmHg, respectively. Treatment with the association of lodenafil + hMSCs reduced RVSP to 29.5 ± 2.2 mmHg, a value similar to the normoxia condition. Heart rate was not affected by PAH (normoxia = 243.3 ± 7.9 bpm, SuHx = 256.0 ± 17.5 bpm) and no statistical difference was observed in heart rate after treatment of lodenafil and hMSCs alone or in combination. No alteration was detected in the mean blood pressure in all experimental groups. Vascular reactivity of the pulmonary artery was affected by PAH, since the ACh-induced relaxation was reduced from 77.5 ± 6.2 to 45.1 ± 8.6% (Figure 2C). Vascular dysfunction was recovered only after treatment with hMSCs in monotherapy (66.9 ± 10.4%) or in association with lodenafil (68.5 ± 3.7%).

### 3.2. Lodenafil + hMSCs Reduces Changes in Lungs from SuHx-PAH Rats

**Figure 3:**
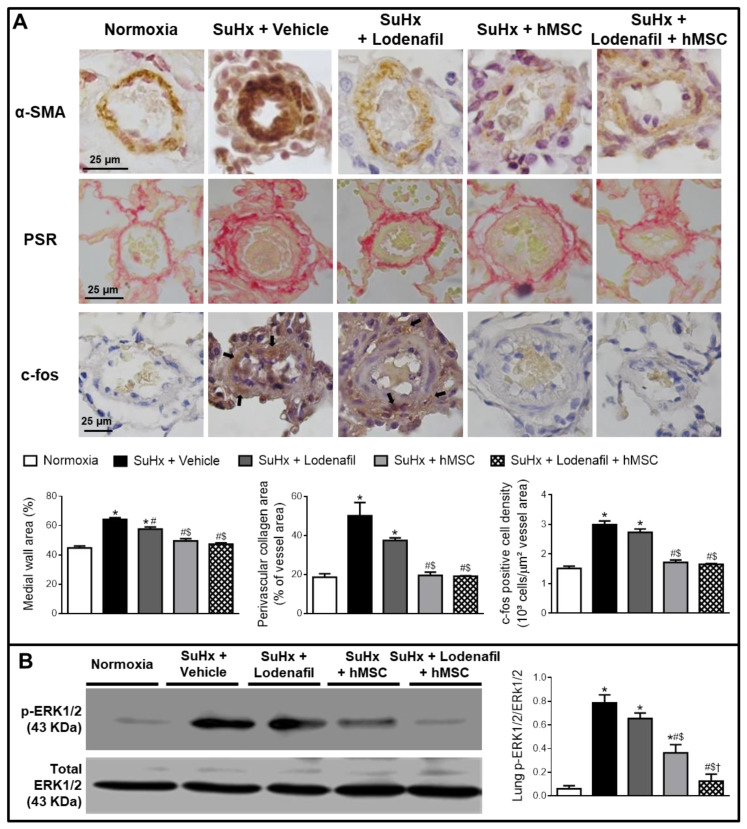
Effects of lodenafil + hMSCs therapy on proliferation markers in lungs from SuHx + PAH rats. (**A**) Representative histological images of pulmonary arterioles (1000× magnification). Black arrows indicate c-fos-stained nuclei of cells. (**B**) Representative images of p-ERK1/2 and total ERK1/2 protein expression in lung homogenates. Data are expressed as mean ± SEM. * *p* < 0.05 compared to normoxia group; # *p* < 0.05 compared to SuHx group treated with vehicle; $ *p* < 0.05 compared to SuHx group treated with lodenafil; † *p* < 0.05 compared to SuHx group treated with hMSCs. One-way ANOVA with multiple comparisons. α-SMA, alpha smooth muscle actin; ERK1/2, extracellular signal-regulated kinase 1/2; hMSCs, human mesenchymal stem cells; PAH, pulmonary arterial hypertension; p-ERK1/2, phosphorylated extracellular signal-regulated kinase 1/2; PSR, picrosirius red; SuHx, SU5416/hypoxia.

**Figure 4:**
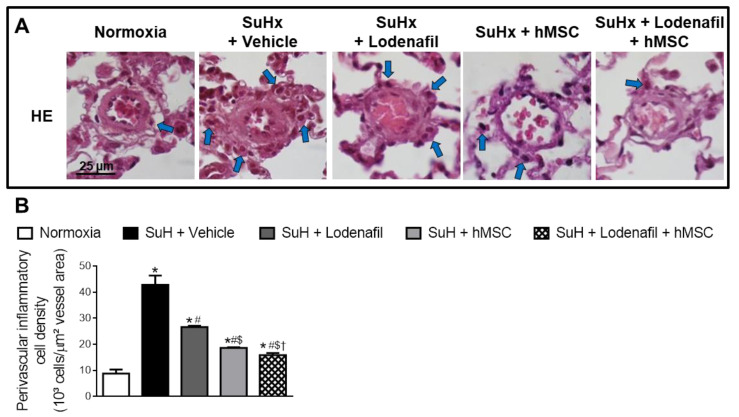
Effects of lodenafil + hMSCs therapy on inflammatory markers in lungs of SuHx + PAH rats. (**A**) Representative histological images of HE staining in pulmonary arterioles. Blue arrows indicate perivascular inflammatory cells. (**B**) Number of perivascular inflammatory cells per pulmonary arteriole area. Data are expressed as mean ± SEM. * *p* < 0.05 compared to normoxia group; # *p* < 0.05 compared to SuHx group treated with vehicle; $ *p* < 0.05 compared to SuHx group treated with lodenafil; † *p* < 0.05 compared to SuHx group treated with hMSCs. One-way ANOVA with multiple comparisons. HE, hematoxylin-eosin; hMSCs, human mesenchymal stem cells; PAH, pulmonary arterial hypertension; SuHx, SU5416/hypoxia.

Hypertrophy of pulmonary arterioles was detected in lungs from the SuHx group because the medial wall area was increased when marked with α-SMA using immunohistochemistry (Figure 3A). In control and PAH animals, the median wall area was of 44.7 ± 1.4 and 64.2 ± 1.2%, respectively. Lodenafil produced an attenuation of this increase (57.6 ± 1.4%) but hMSCs in monotherapy and associated to lodenafil reversed the hypertrophy of the pulmonary wall (47.3 ± 0.9%). PAH increased the perivascular collagen deposit in pulmonary arterioles from 18.6 ± 1.8 to 50.1 ± 6.7% and this condition was not normalized after lodenafil treatment. In contrast, when lodenafil was associated with hMSCs, it reversed to 19.1 ± 0.3%. c-Fos immunostaining in pulmonary arterioles from hypoxic animals showed an increase of 3-fold in the marked cell density and treatment with lodenafil + hMSCs reduced from 3.0 ± 0.1 × 10^3^ to 1.7 ± 0.1 × 10^3^ cells/mm^2^. The p-ERK1/2 per total ERK1/2 expression ratio seen in lungs from control animals was of 0.06 ± 0.03 (Figure 3B). PAH increased that ratio to 0.79 ± 0.07, which was not altered by lodenafil (0.65 ± 0.05) but was totally reversed to 0.12 ± 0.06 when animals were treated with lodenafil + hMSCs. Representative images of pulmonary arterioles stained with HE are shown in Figure 4A. The number of perivascular cells per vessel area in control rats was 8.8 ± 1.5 × 10^3^ cells/μm^2^ (Figure 4B). In the PAH condition, the value was increased to 42.8 ± 3.5 × 10^3^ cells/μm^2^ which was partially reverted by lodenafil (26.6 ± 0.4 × 10^3^ cells/μm^2^). Therefore, when lodenafil was associated with hMSCs, it showed a further reduction to 15.8 ± 0.8 × 10^3^ cells/μm^2^.

### 3.3. Lodenafil + hMSCs Therapy Reverses Structural Changes in RV from SuHx-PAH

**Figure 5:**
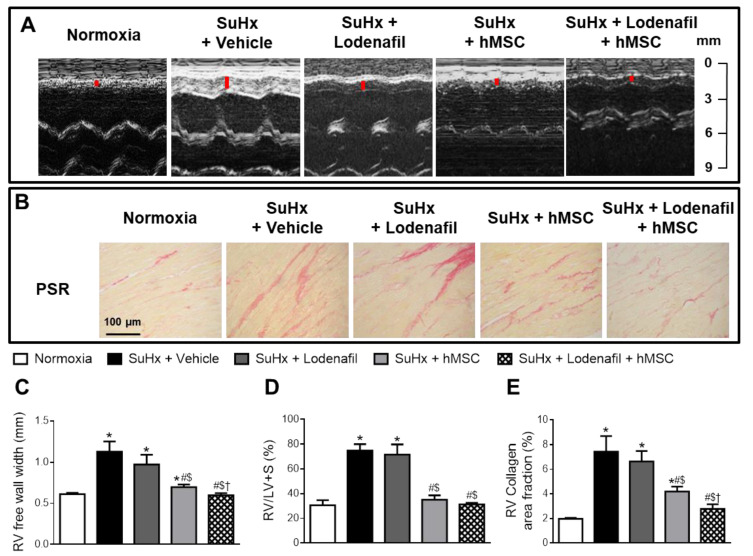
Effects of lodenafil + hMSCs therapy on proliferation markers in the RV from SuHx + PAH rats. (**A**) Representative images of the RV free wall width, obtained using M-mode echocardiography. (**B**) Representative images of PSR staining in RV fields (400× magnification). (**C**) RV free wall width. (**D**) RV/LV+S ratio (Fulton’s index). (**E**) RV collagen area fraction. Data are expressed as mean ± SEM (*n* = 6). * *p* < 0.05 compared to normoxia group; # *p* < 0.05 compared to SuHx group treated with vehicle; $ *p* < 0.05 compared to SuHx group treated with lodenafil; † *p* < 0.05 compared to SuHx group treated with hMSCs. hMSCs, human mesenchymal stem cells; LV, left ventricle; PAH, pulmonary arterial hypertension; PSR, picrosirius red; RV, right ventricle; S, septum; SuHx, SU5416/hypoxia.

Figure 5A shows representative images of the RV free wall width using M-mode echocardiography. HAP induced an increase in the RV free wall width from 0.61 ± 0.02 to 1.13 ± 0.12 mm (Figure 5C), indicating RV hypertrophy. Lodenafil treatment did not alter this change (0.97 ± 0.12 mm), but its association with hMSCs resulted in a reversal to 0.60 ± 0.03 mm. An increase at the RV/LV + S weight ratio was observed due to PAH, altering from 30.6 ± 4.1 to 74.8 ± 5.1% (Figure 5D). Lodenafil alone had no effect over this parameter, but it reversed after hMSCs therapy alone (35.1 ± 3.5%) or in association with lodenafil (31.3 ± 1.4%). RV collagen deposition was intensified by PAH (Figure 5B), increasing from 1.97 ± 0.08 to 7.44 ± 1.25% (Figure 5E) and the association lodenafil + hMSCs reduced to 2.78 ± 0.38%.

### 3.4. Lodenafil + hMSCs Therapy Reverses Increased Inflammatory Markers in the RV from SuHx-PAH

**Figure 6:**
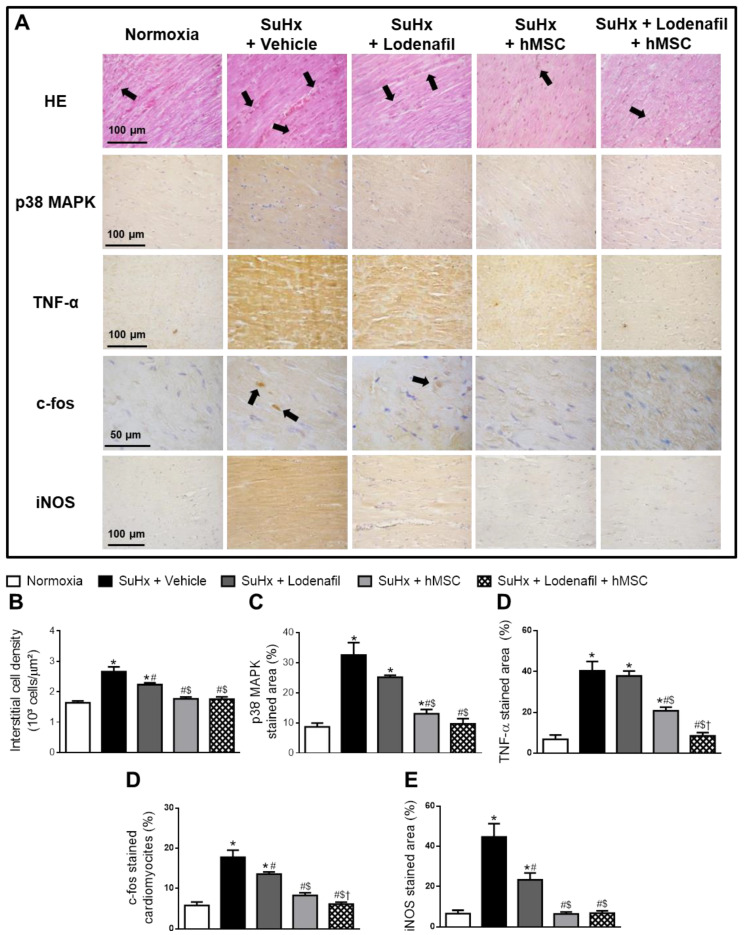
Effects of lodenafil + hMSCs therapy on inflammatory markers in the RV from SuHx + PAH rats. (**A**) Representative images of histological and immunohistochemical stainings in RV fields. (**B**) Number of interstitial cells per μm^2^ in RV tissue area. (**C**) RV tissue area positively stained with p38 MAPK. (**D**) RV tissue area positively stained with TNF-α. (**E**) Number of cardiomyocytes positively stained with c-fos. (**F**) RV tissue area positively stained with iNOS. Data are expressed as mean ± SEM (*n* = 6). * *p* < 0.05 compared to normoxia group; # *p* < 0.05 compared to SuHx group treated with vehicle; $ *p* < 0.05 compared to SuHx group treated with lodenafil; † *p* < 0.05 compared to SuHx group treated with hMSCs. HE, hematoxylin-eosin; hMSCs, human mesenchymal stem cells; iNOS, inducible nitric oxide synthase; MAPK, mitogen-activated protein kinase; PAH, pulmonary arterial hypertension; RV, right ventricle; SuHx, SU5416/hypoxia; TNF-α, tumor necrosis factor alpha.

SuHx led to an increased interstitial cell density in the RV from hypoxic animals, as seen in the HE staining images (Figure 6A). Interstitial cell density values in control and PAH animals were 1.64 ± 0.06 and 2.66 ± 0.15 × 10^3^ cells/μm^2^, respectively (Figure 6B). Lodenafil attenuated this inflammatory component, reducing to 2.23 ± 0.06 × 10^3^ cells/μm^2^, while treatment with hMSCs alone and associated with lodenafil lowered to values similar to normoxia of 1.77 ± 0.06 and 1.76 ± 0.08 × 10^3^ cells/μm^2^, respectively. p38 MAPK content in the RV from control animals was 8.7 ± 3.1% (Figure 6C), which was increased about 4-fold by PAH. While lodenafil treatment did not reverse this change, treatment with lodenafil + hMSCs led to a reversal to 9.6 ± 1.8%. PAH also increased TNF-α content in the RV, since the TNF-α-stained area increased from 6.8 ± 2.1 to 40.4 ± 4.5% (Figure 6D). Reversal to 8.5 ± 1.7% was observed after treatment with lodenafil + hMSCs but not with monotherapy of lodenafil. Cardiomyocytes with nuclei positively stained for c-fos compared to the total amount of cells in the RV revealed an increase from 5.8 ± 0.9 to 17.8 ± 1.8% due to PAH which was partially reversed by lodenafil (13.6 ± 0.5%) and normalized by lodenafil + hMSCs (6.1 ± 0.5%). The iNOS-stained area analysis in RV fields showed an increase from 6.5 ± 1.7 to 44.7 ± 6.6 when comparing in PAH animals (Figure 6E). Lodenafil partially reversed to 23.3 ± 3.4%, while hMSCs treatment alone or in association with lodenafil had it brought down to values similar to the normoxia group of 6.5 ± 0.9 and 6.8 ± 1.1%, respectively.

## 4. Discussion

UC-hMSCs (5.10^5^ cells i.v.) therapy was previously reported to reverse the alterations induced by PAH in mice [14]. This treatment was also successful to interfere with other disease models, such as stroke, in which a high amount of cells was required to improve cerebral dysfunction [26]. In the present work, a lower number of cells in combination with a PDE5 inhibitor was administered, which could reduce the risk of complications related to stem cell therapy, such as pulmonary embolism [27]. The main purpose was to demonstrate the benefit provided by this association in hypoxia-induced PAH in rats, using a sub-therapeutic dose of lodenafil [11,24], with the expectation to identify possible additional effects to hMSCs therapy without causing severe systemic adverse effects. Lodenafil alone or in association with hMSCs did not alter systemic blood pressure, reinforcing the safety of this therapeutic approach.

A single administration of hMSCs was used, followed by short-term therapy of lodenafil, which could be the explanation for the recovery of not all structural and functional properties of the heart and lungs. Partial reversal of PAH characteristics was detected only after six months when a high number of bone marrow-derived MSCs were tested in a monocrotaline-induced PAH model [28]. In the model of SuHx-induced PAH, a long-term observation would be inappropriate by the fact that partial spontaneous recovery could be detected on the PAH phenotype after months [29].

Anti-proliferative and anti-inflammatory effects induced by hMSCs could recover the pulmonary artery function and structure, and endothelial integrity. The paracrine factors released by hMSCs could produce direct vasodilation in the pulmonary vasculature which was previously demonstrated to ameliorate several ultrastructure changes caused by MCT-induced PAH, such as elastic fiber discontinuations in the intima and increasing serum NO levels (Chen et al., 2017) [30].

**Figure 7:**
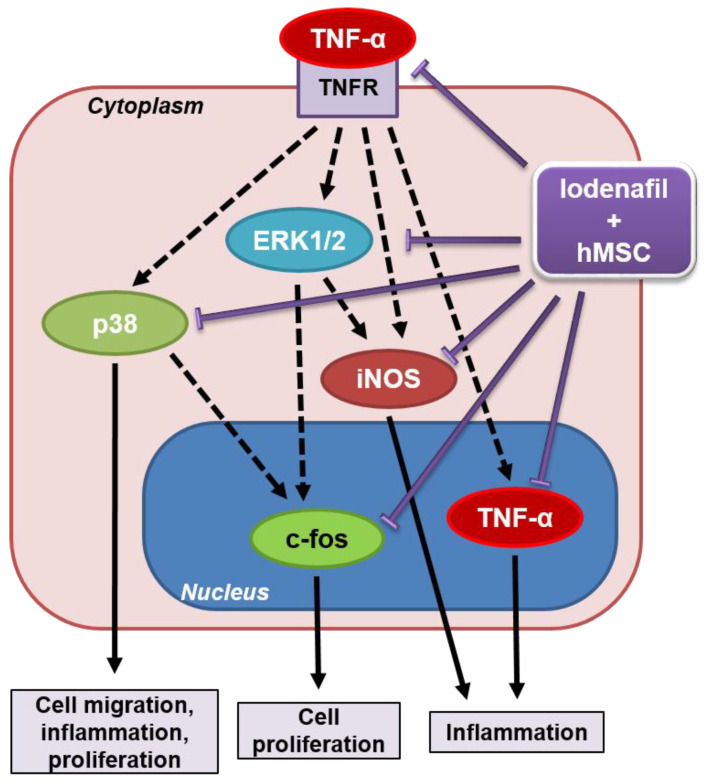
Schematic representation of molecular sites involved in the action of lodenafil + hMSCs association in SuHx-PAH rats. Black arrows indicate the effects promoted by the shown proteins in SuHx-PAH rats, while purple arrows indicate protein expression reduction in lung and RV induced by lodenafil and hMSCs therapy. ERK1/2, extracellular signal-regulated kinase 1/2; hMSCs, human mesenchymal stem cells; iNOS, inducible nitric oxide synthase; p38, p38 mitogen-activated protein kinase; SuHx, SU5416/hypoxia; TNF-α, tumor necrosis factor alpha; TNFR, TNF-α receptor.

Lodenafil + hMSCs altered the expression of proteins involved in cellular proliferation and inflammation related to PAH, indicating the anti-proliferative and anti-inflammatory effects (Figure 7). Functional and structural improvement suggested that both pulmonary arteriole and RV remodeling were reverted by the association because of the reduction in perivascular and RV fibrosis.

MSCs release several paracrine mediators, which could interfere with the evolution of PAH [15,31]. These include cytokines, chemokines, growth factors, microRNAs (miRNA) and extracellular vesicles [16]. Two miRNA released by MSCs that have been linked to an anti-proliferative effect in HAP are miRNA-204 [32] and miRNA-let-7a [33]. An anti-inflammatory effect induced by MSCs is associated mainly with the reduction in inflammatory cytokine interleucin 6 (IL-6) expression and CD68+ macrophages in the lung [34].

A downstream signaling molecule related to the proliferation phenotype in PAH is c-fos, whose expression is increased at pulmonary arterioles and is responsible for enhanced pulmonary artery smooth muscle cell proliferation in an animal model and in PAH patients [35]. Since c-fos proliferation in pulmonary artery smooth muscle exposed to hypoxia depended on miRNA-214-3p [36], hMSCs could regulate local miRNA to promote an anti-proliferative effect. It is possible that miRNA-214-3p could be downregulated by lodenafil + hMSCs because of the reversal of increased c-fos expression in pulmonary arterioles. Both miRNA-204′s and miRNA-let-7a’s anti-proliferative properties have been related to their suppressing of the IL-6/STAT3 signaling pathway, a central determinant of the hyperproliferative vascular cell phenotype in patients with idiopathic PAH (Lee et al., 2012; Chen et al., 2017). miRNA-214-3p, on the other hand, is upregulated in response to signaling by TGF-β1, another critically important mediator of pathophysiological events in PAH and fibrosis (Stevens at al., 2016). Recent evidence showed that TGF-β1 can also upregulate STAT3 in PAH (Cai et al., 2018), suggesting that there might be a relationship between released miRNA-204 and miRNA-let-7a and a decrease in miRNA-214-3p, which would be reflected in the reversal of increased c-fos expression.

ERK1/2 is a member of the MAPK superfamily and a potent regulator of cell growth that can be activated by various extracellular stimuli, such as mitogens, growth factors and cytokines [37], and it is overexpressed in endothelial cells of PAH patients [38]. Lodenafil + hMSCs intensively reduced expression of this protein in the lungs, suggesting that lodenafil could improve the anti-proliferative activity of hMSCs on pulmonary arterioles. In contrast, pulmonary artery wall hypertrophy and perivascular fibrosis were not altered by lodenafil alone or in association with hMSCs. The lack of effect may be due to the short-term treatment, in which the association improved the molecular profile but not the structure of tissues.

p38 MAPK is another member of the MAPK superfamily that is upregulated in PAH, whose activation is correlated with fibroblast-to-myofibroblast differentiation by pathways dependent on TGF-β1 and TRPC6 [39], and increased collagen synthesis and depositing by RV fibroblasts [40]. Increased p38 MAPK levels in PAH patients are also associated with increased levels of inflammation markers whose inhibition led to their reduction [41]. p38 MAPK inhibition improves RV function and reverses fibrosis through the reduction of collagen content and production by cardiac fibroblasts in the RV [40]. The SuHx model of PAH led to an increase in p38 MAPK content in RV tissue, which was reversed by lodenafil + hMSCs, suggesting a beneficial effect over RV remodeling through an anti-inflammatory and anti-fibrotic mechanism.

TNF-α is a pro-inflammatory mediator whose serum level is elevated in PAH patients [42]. Besides promoting inflammatory responses such as cell migration, macrophage activation and apoptosis [43], TNF-α is correlated with the remodeling of the pulmonary vasculature in PAH, by activation of MAPK-dependent proliferative pathways [43] and induction of the endothelium–mesenchymal transition [44]. Besides its deleterious effects in the pulmonary vasculature in PAH, increased TNF-α is involved in RV remodeling (chamber dilatation and fibrosis) and its altered contractility, leading to the development of RV failure [45]. The increase in TNF-α expression in the RV observed in hypoxia-induced PAH is not altered by lodenafil in monotherapy. However, when lodenafil was associated with hMSCs, it reversed the TNF-α expression increase, indicating that the association promoted a cardioprotective effect through an anti-inflammatory mechanism.

Endothelin-1 signaling promotes cardiac hypertrophy through c-fos-dependent pathways [46]. Alterations of the endothelin pathway and increased density of ET_A_ receptors are detected in the RV of PAH patients [47]. An increase in cardiac c-fos levels is also seen in angiotensin II-induced inflammatory damage in the LV [48], and considering that an increase in angiotensin II signaling is observed in the RV during PAH [49], it is possible to consider that c-fos is related to inflammatory pathways in RV remodeling and dysfunction in PAH. Lodenafil + hMSCs reduced the expression of c-fos in cardiomycytes, indicative of anti-proliferative and anti-inflammatory properties.

iNOS is associated with inflammatory cytokine effects in the heart leading to RV failure in monocrotaline-induced PAH animals [50]. ERK1/2-dependent iNOS activation in cardiac fibroblasts is involved in the decrease in L-type Ca^2+^ channel activity in RV cardiomyocytes, which is responsible for cardiac dysfunction [51]. Lodenafil alone promoted a partial reversal of increased iNOS levels in the RV, while its association with hHSCs normalized its expression, which could explain the improvement of cardiac dysfunction.

## 5. Conclusions

Combination therapy of lodenafil and hMSCs reversed the functional, structural and molecular changes induced by SuHx-PAH through anti-inflammatory and anti-proliferative action, and may be an alternative therapeutic strategy for PAH treatment.
